# An Airborne Offner Imaging Hyperspectrometer with Radially-Fastened Primary Elements

**DOI:** 10.3390/s20123411

**Published:** 2020-06-17

**Authors:** Nikolay Kazanskiy, Nikolay Ivliev, Vladimir Podlipnov, Roman Skidanov

**Affiliations:** 1 Image Processing Systems Institute of RAS—Branch of the Federal Scientific Research Centre “Crystallography and Photonics” of Russian Academy of Sciences, 151 Molodogvardeyskaya st., 443001 Samara, Russia; kazanskiy@ssau.ru (N.K.); ivlievn@gmail.com (N.I.); 2 Department of Technical Cybernetics, Samara National Research University, Moskovskoe shosse34, 443086 Samara, Russia; podlipnovvv@ya.ru

**Keywords:** imaging hyperspectrometer, Offner scheme, thermal stability

## Abstract

We propose a new layout for the Offner imaging hyperspectrometer that is utilized onboard small space vehicles. The layout is based on a method of adjusting the adaptive temperature-dependent optical scheme by moving just two coaxial optical components located inside the hyperspectrometer. We present the results of modeling for a temperature range of −40 to +45 °C and an optical experiment using a heat and cold chamber for the range of 12 to 40 °C, proving the basic functionality of the proposed layout. Based on simulation results, the new layout is supposed to allow the hyperspectrometer to operate in a temperature range of −40 to +45 °C without its optical characteristics deteriorating, thus making it suitable for work onboard space or stratospheric vehicles.

## 1. Introduction

An Offner scheme was described for the first time in Reference [1] as an optical setup for projection lithography. This optical scheme eliminates third- and fifth-order spherical aberration, third- and fifth-order coma, and third-order astigmatism. As a result, the only remaining significant geometric aberration is fifth-order astigmatism [1,2,3]. Subsequently, the optical scheme was applied to solve a wide range of problems [4,5], including determining the wavelength of light, where it was combined with a Michelson interferometer.

High-quality imagery acquired with the use of the Offner scheme prompted the idea to utilize it in an imaging spectrometer [6]. With a diffraction grating synthesized on the secondary convex mirror (to decompose light into a spectrum), we got a spectral device with a minimum level of distortion aberration. However, with the diffraction grating introducing changes in the angles of light propagation, off-axis aberrations ceased to be fully compensated for. There are quite a few publications dealing with different ways of reducing the level of off-axis aberrations [3,4,6,7,8,9,10,11,12,13,14,15,16].

The main application area for imaging spectrometers is in Earth remote sensing (ERS), and Offner imaging spectrometers have been developed for the same purpose [7]. An imaging spectrometer mounted onboard an atmospheric or space vehicle experiences fairly strong thermal deformations, which affect both optical elements and the mounting hardware for fastening and aligning the hyperspectrometer. In Reference [7], the impact of thermal deformations on the elaborate optomechanical design for fastening optical elements was analyzed. The solution proposed in that study relied on the use of alloys with low thermal expansion and dedicated heating elements to actively regulate the temperature of the device. However, such an approach also suffers from a number of shortcomings, such as increased energy consumption and the need to accurately control the level of heating.

In this work, we propose a new layout of the optomechanical system of an Offner imaging spectrometer with a dispersive element that was discussed in References [17,18,19,20,21]. We numerically evaluate the effect of thermal deformations on the image quality and analyze the results of an optical experiment with different temperatures of the hyperspectrometer. Based on the results of the computer modeling and optical experiment, we infer that precise thermal adjustment of the hyperspectrometer can be made by moving two components.

## 2. Materials and Methods

**Figure 1:**
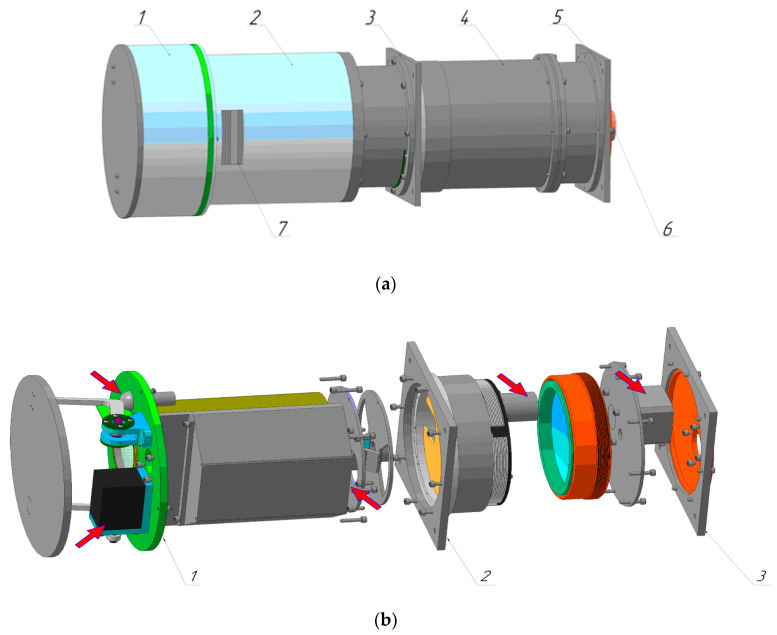
Appearance of hyperspectrometer: (**a**) with external housing: *1*: cover unit, *2*: case to protect the objective lens and fasten the cover unit to the hyperspectrometer, *3* and *5*: brackets for fastening to a space vehicle, *4*: hyperspectrometer case, *6*: connector for plugging in thermal and terminal sensors, *7*: hatch for connector access, and (**b**) without external housing: *1*, *2*, *3*: units for fastening the spectrometer to a space vehicle. Mechanisms for optical calibration and moving the mirror and diffraction grating are shown by arrows.

We propose a design of the Offner imaging hyperspectrometer that contains two concentric (coaxial) optical elements. A distinctive feature of the hyperspectrometer layout proposed herein is that instead of fastening elements to the main platform, all primary optical elements are fastened to a tubular housing using axisymmetric clamps (Figure 1). Clamps of two elements, the primary mirror of the spectral unit and the diffraction grating, are located on the female thread of the tubular housing. This makes it possible to perform high-precision on-axis alignment of the elements. At the same time, thanks to the use of the proposed scheme for optical element fastening, variations in the element tilt relative to the optical axis can be minimized.

Putting aside gradient and transient processes, temperature variations are linked with the thermal expansion of materials, causing changes in both the distance between optical elements and the optical parameters of the elements (curvature radii).

Airborne and spaceborne hyperspectrometers normally operate in a standard temperature range of −40 to +45 °C. Considering that the optical circuit of the Offner imaging hyperspectrometer discussed herein is intended for operation at 20 °C, we needed to analyze a situation where the temperature drops by 60 °C and rises by 25 °C. Using the simplest thermal deformation model, we can easily show that the curvature radius of spherical surfaces is proportional to the linear expansion.

### 2.1. Optical Modeling

The optical modeling was conducted using a commercial program, Zemax.

Table 1 gives the major parameters of the hyperspectrometer’s optical elements.

A binary diffraction grating with a 10 µm period was synthesized on the surface of a convex mirror to enable operation in the negative-first order of a shortwave infrared (SWIR) array.

**Figure 2:**
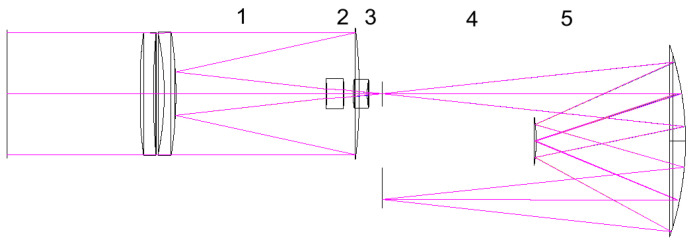
Essential segments of the optical scheme of the hyperspectrometer with an objective.

In this section, we analyze how temperature variations affect the imaging quality of the hyperspectrometer. For this purpose, the optical scheme of the hyperspectrometer was broken down into five essential segments whose varied length could have a significant impact on the imagery (Figure 2). Segments 1 and 2 denote distances between the optical elements in the objective, segment 3 denotes the distance from the end surface of the objective to a slit diaphragm, segment 4 denotes the distance from the plane of the slit diaphragm to the Offner’s primary mirror, and segment 5 denotes the distance between movable concentric mirrors.

Table 2 gives the basic lengths of the segments at a temperature of 20 °C.

In the modeling, the lenses and mirrors were assumed to be made of fused silica (thermal expansion coefficient, α = 1.4 × 10^−6^ K^−1^) and the housing of an aluminum alloy, D16T (thermal expansion coefficient, α = 2.2 × 10^−5^ K^−1^). Unfortunately, the standard temperature variation option in Zemax was fundamentally unable to account for the housing properties, so variations in the distance between elements had to be calculated separately and added to the optical scheme parameters manually.

The optical modeling was conducted in two stages. First, we derived a modulation transfer function (MTF) of the hyperspectrometer objective, then the MTF of the entire hyperspectrometer.

### 2.2. Optical Experiment

**Figure 3:**
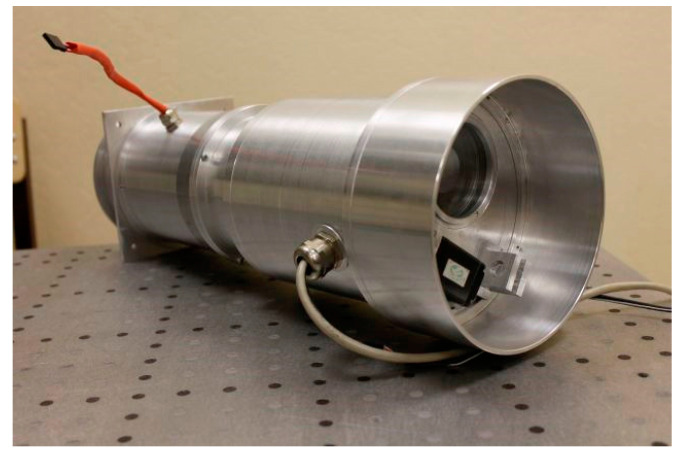
External appearance of prototype hyperspectrometer.

A prototype hyperspectrometer was fabricated in exact compliance with the design parameters in Table 1. As a photosensitive array, the CMV4000 with 5.5 µm pixels was utilized. Figure 3 shows the external appearance of the hyperspectrometer without a calibration unit, which is not required for a laboratory experiment.

**Figure 4:**
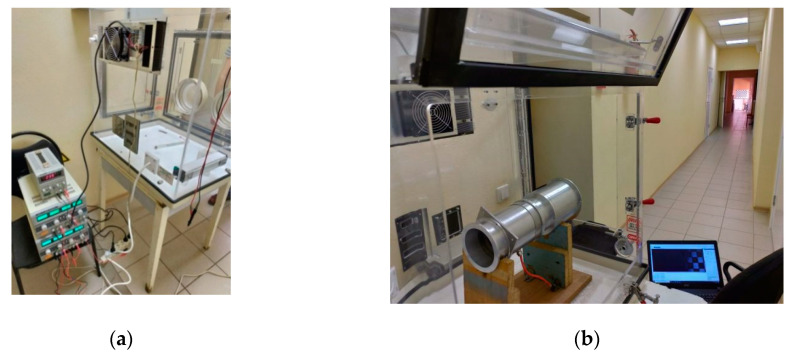
(**a**) Transparent box with temperature control function and (**b**) position of hyperspectrometer during the experiment.

To change its temperature, the hyperspectrometer was placed in a transparent box equipped with a heater and a Peltier refrigerator (Figure 4).

Since it has a long-focus objective, the hyperspectrometer and a point light source were separated by 20 m (Figure 4b). The point source was in the form of a circular 1.4 mm pinhole in a screen that was backlit by a 633 nm He–Ne laser. The point source was located so as to make an angle of 1.2° with the optical axis in the visual field, in compliance with optical modeling parameters. The device was initially calibrated at 20 °C, before being heated to 40 °C and then cooled to 12 °C (we were unable to reach lower temperatures).

## 3. Results

### 3.1. Modeling the Hyperspectrometer Objective

**Figure 5:**
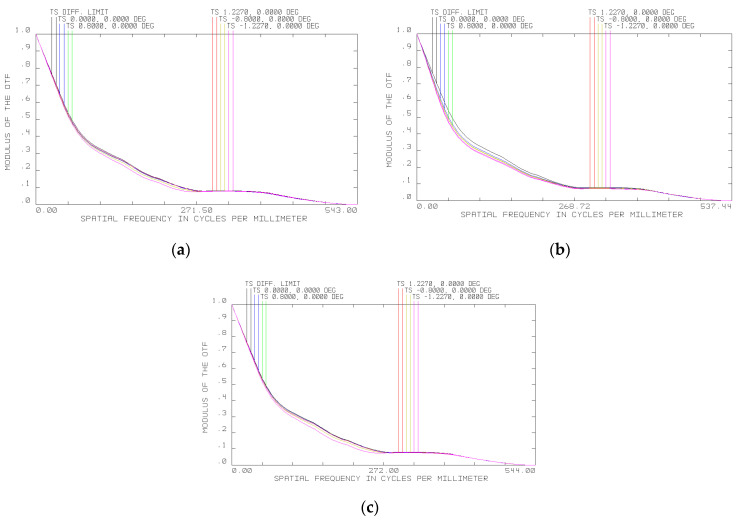
Modulation transfer function (MTF) of the objective at (**a**) 20 °C, (**b**) −40 °C, and (**c**) 45 °C.

The modulation transfer function of the objective at 20, −40 and 45 °C is shown in Figure 5a–c, respectively. The modeling shows that at −40 °C, the focal length of the objective changes by 2.9 mm. This change can be compensated for by shifting the entire spectral unit by 1.45 mm (the value of the working segment variation). When the objective is heated to 45 °C, the objective parameters change somewhat less. If changes in the objective focal length are taken into account and the registration plane position is changed, the MTF of the objective changes insignificantly with changing temperature. At 45 °C, the change of the focal length is just 0.54 mm, making it possible to compensate for image distortions by moving the whole spectral unit by 0.27 mm.

Hence, the required adjustment is just 1.72 mm. Below, we analyze the modulation transfer function (MTF) in a frequency range up to 200 lines/mm, which is close to the spatial recording frequency limit of modern photosensitive arrays (with a 2.5 µm photosensitive cell). Our hyperspectrometer utilizes an array with large-size photosensitive elements, enabling recording in a high-frequency range (about 300 Hz), thus doing without pitch during orbital motion.

### 3.2. Modeling a Spectral Unit of the Hyperspectrometer

**Figure 6:**
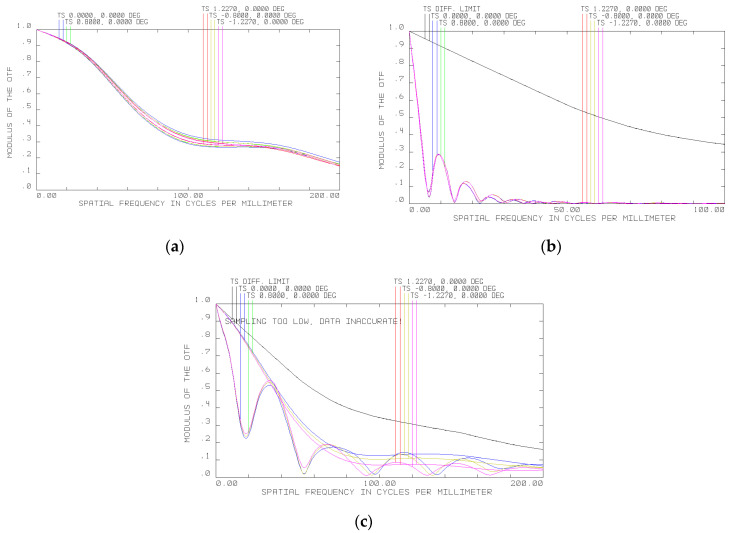
MTF of hyperspectrometer at (**a**) 20 °C, (**b**) −40 °C, and (**c**) 45 °C.

The MTF of the hyperspectrometer at 20, −40 and 45 °C is shown in Figure 6a–c, respectively. Changes in total MTF due to thermal deformations of the spectral unit elements are much more essential.

**Figure 7:**
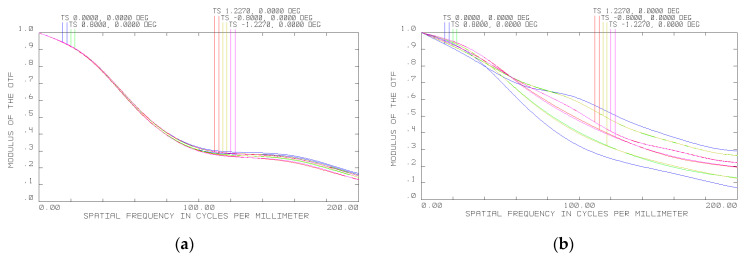
MTF of hyperspectrometer following spectral unit adjustment relative to photosensitive array at (**a**) −40 °C and (**b**) 45 °C.

It can be seen from Figure 6 that the MTF becomes essentially narrower. However, in general, such deterioration can easily be compensated for by shifting the optical elements of the spectral unit relative to the photosensitive arrays. Specifically, the primary mirror and diffraction grating need to be moved, respectively 1.45 and 0.73 mm away from the objective at −40 °C and 0.27 and 0.135 mm toward the objective at +45 °C. Figure 7 shows the results of optical modeling of the MTF following the necessary shifts of the hyperspectrometer’s spectral unit elements to compensate for thermal deformations. The MTF is seen to be again close to the diffraction limit (Figure 7a,b).

Thus, we can infer that it is necessary to use an adjustment mechanism to shift two mirrors of the spectral unit, with a maximum shifting range of 2.56 mm for each element.

**Figure 8:**
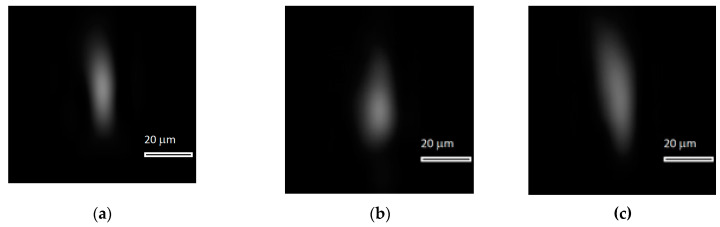

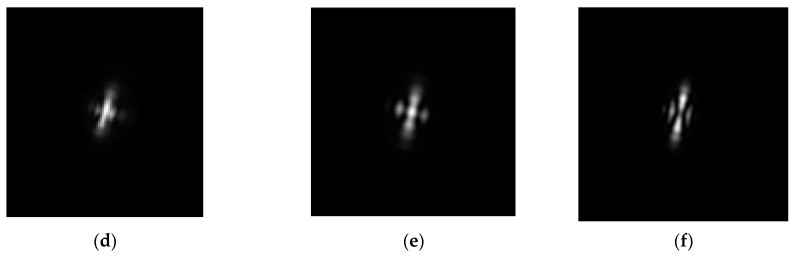
Experimentally derived point spread functions (PSFs) (interpolated using 5.5 × 5.5 µm pixels) at (**a**) 20 °C, (**b**) 40 °C, and (**c**) 12 °C, and (**d**–**f**) PSFs numerically calculated in the Zemax program for the same temperatures

Based on optical modeling results, values of necessary shifts for two optical elements (mirror and diffraction grating) were derived for a range of temperatures from −40 to +45 °C with a step of 5 °C (Table 1). Figure 8d–f depicts point spread function (PSF) images modeled on the photodetector at 20, 40 and 12 °C after the spectral unit and objective were adjusted to compensate for thermal deformations in accordance with the values in Table 3.

Using the data from Table 3, the hyperspectrometer can be automatically adjusted according to accurate temperature values.

### 3.3. Experimental Characterization of Hyperspectrometer

Figure 8a–c depicts experimental PSFs at 20, 40 and 12 °C respectively, which were derived after the positions of the two hyperspectrometer elements were adjusted by the values given in Table 3. For comparison, Figure 8d–f shows the PSFs calculated for the same temperatures using Zemax.

**Figure 9:**
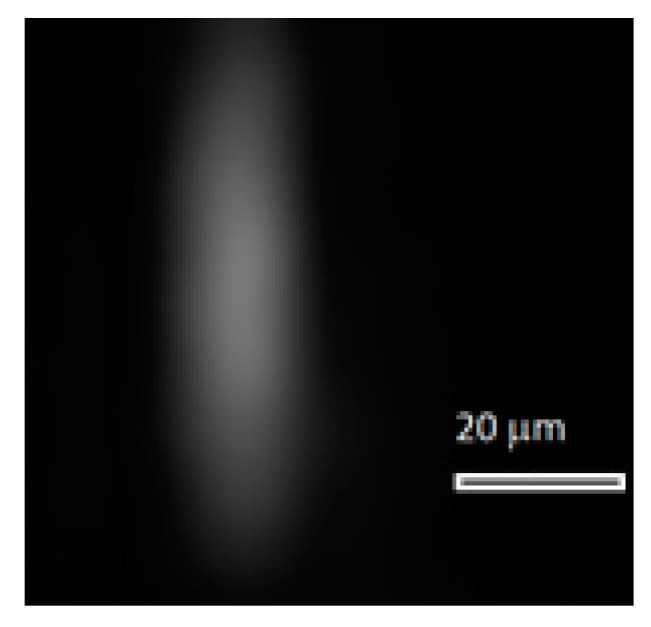
Experimentally measured point spread functions (PSFs; interpolated by 5.5 × 5.5 µm pixels) at 35 °C without optical scheme adjustment.

Intensity distributions in Figure 8 are elongated along the perpendicular direction to the diffraction grating grooves. In this direction, the PSF width at full-width half-minimum (FWHM) intensity is 22 µm for each intensity distribution (four pixels of the array). Along the grating grooves, the FWHM width is 11 µm (two pixels of the array). Thus, the PSF width remains practically unchanged in a fairly wide range of temperatures, which is also confirmed by the modeling results. For comparison purposes, Figure 9 presents an experimental PSF at 35 °C without adjustment. The PSF width is 33 µm (six pixels) measured perpendicular to the grating grooves, amounting to 16.5 µm (three pixels) measured along the grooves.

## 4. Discussion

An analysis of the hyperspectrometer MTF at different temperatures (Figure 7) suggests that it has an appropriate level for obtaining high-quality imagery in a temperature range of −40 to +45 °C when using recording frequencies up to 200 mm^−1^ (above 0.15). For the photodetector utilized in the prototype hyperspectrometer, the MTF level is higher than 0.3 in the same temperature range. As one would expect, the adjustment value turned out to be smaller for increasing temperatures, but this is because there is less deviation from the normal working temperature of 20 °C. The range of −40 to +45 °C is a working range of the recording photodetector, so it makes no sense to study the prototype beyond this temperature interval. At first sight, the experimental data are different from those obtained by the optical modeling in Zemax; however, one should bear in mind that the image interpolated in Figure 8 and Figure 9 has a width of just two pixels. Considering that the PSF width together with side lobes modeled in Zemax is 11 µm, it should be represented by only two photodetector pixels, meaning that it is impossible to obtain a more accurate image. Thus, the modeling and experimental results may be considered to be in good agreement.

The element position adjustment is implemented using two compact step motors, which have an essential benefit in both mass (by an order of magnitude) and energy consumption over a thermo-stabilization system that is comparable in mass with the total mass of the hyperspectrometer. In the optical experiment, the range of temperatures was studied only partially. The Peltier refrigerator’s power was not high enough to cool the device below 12 °C, while heating above 40 °C was associated with a risk of damaging the recording photodetector. Nonetheless, in the experimentally studied range of temperatures, the calculated adjustment value made it possible to restrict the PSF to a standard size, and we can suggest that the layout will also work at other temperatures. Thus, the use of the hyperspectrometer layout proposed in this work actually makes it possible to do without a thermo-stabilization unit, while preserving the imagery quality. Moreover, the optical scheme can be scaled up or down because all the changes studied are nearly linear, so that an essentially more compact hyperspectrometer can be designed. As demonstrated in Reference [22], using the Offner scheme, the upper bound of the recorded spectral range can be extended practically without optical characteristics deteriorating close to 3 µm (a wavelength). In this case, the configuration of spectral channels remains linear, unlike, say, in a hyperspectrometer based on a complex prism [23].

## 5. Conclusions

The scientific novelty of the presented hyperspectrometer in terms of the dispersion element, operation features, and calibration is well represented in References [18,19,20,24]. However, without the results presented in this work, this hyperspectrometer would have remained a laboratory model. The results of the study presented in this paper allow the hyperspectrometer to become a real instrument for space- and aviation-based applications.

The analysis conducted on the basis of the optical modeling and optical experiment shows that with a radial layout of fasteners of optical elements, it will suffice to have just two movable elements in the optical system to operate the hyperspectrometer without image quality deterioration in the temperature range of −40 to +45 °C. The objective’s MTF undergoes no essential variation when working in the indicated range. Results of the optical experiment were obtained in the range from 12 to 40 °C. The PSF width measured in this range was found to remain unchanged (about 11 µm). The result obtained allows us to make a well-grounded assumption that the Offner imaging hyperspectrometer can operate with high efficiency in a wide range of temperatures without the use of a thermo-stabilization system to maintain temperature. The proposed approach shows promise for space and stratospheric use.

## Figures and Tables

**Table 1:** Major parameters of Offner imaging hyperspectrometer.

Element	Radius 1 (mm)	Radius 2 (mm)	Thickness (mm)	Diameter (mm)	Material
Lens 1	247	395	7.34	65	Silica
Lens 2	–162	–215	6.17	65	Silica
Major objective mirror	–300	–	10	70	Silica
Minor objective mirror	–215	–	6,17	28.7	Silica
Lens 3	–75	–120	9	16	Silica
Lens 4	42.7	39.5	7.5	15	Silica
Major spectrometer mirror	–159.64	–	20	90	Silica
Diffraction grating	–80.6	–	10	25.4	Silica

**Table 2:** Basic lengths of segments between scheme components at a temperature of 20 °C.

Segment	1	2	3	4	5
Length (mm)	96.7	4.79	7	160	78.72

**Table 3:** Shift values for mirror and spherical diffraction grating at different temperatures.

Temperature (°C)	Mirror Shift (µm)	Spherical Reflection Diffraction Grating Shift (µm)
−40	1450	730
−35	1330	670
−30	1210	610
−25	1090	550
−20	970	490
−15	850	430
−10	725	360
−5	605	300
0	480	240
5	360	180
10	240	120
15	120	60
20	0	0
25	−54	−25
30	−110	−55
35	−160	−80
40	−220	−110
45	−270	−135
